# The effects of working memory training in children revealed by behavioral responses and ERP

**DOI:** 10.1002/brb3.2310

**Published:** 2021-08-01

**Authors:** Jie Xu, Meiqi Deng, Wenya Nan, Dan Cai

**Affiliations:** ^1^ Department of Psychology Shanghai Normal University Shanghai China; ^2^ School of Foreign Languages in Tourism Shanghai Institute of Tourism Shanghai China

**Keywords:** children, cognitive training, event‐related potentials, neurophysiological outcomes, working memory

## Abstract

**Background:**

Recent studies have examined the effect of computerized cognitive training on working memory (WM), but the behavioral and neural effects were uncertain. Also, few studies have explored WM training effects on children using event‐related potentials. The purpose of our study was to investigate the effects of WM training in children, including the effects on behavioral performance and neurophysiological outcomes.

**Methods:**

Forty‐four healthy children (mean age = 7.76 years, *SD *= 0.57 years, 18 females) were assigned to the training and control groups. Over 20 training sessions, the training group participated in the computation‐span and spatial N‐back tasks, whereas the control group joined in normal class activities. They all completed the pre‐ and post‐test evaluation of WM tasks (digit span backwards task and N‐back task).

**Results:**

The results showed that WM training led to improved performance in the digit span backwards task and 2‐back task of post‐test evaluation, shortened P3a and P3b latencies in nontarget trials during the spatial 1‐back task, shortened P3a latency in target and nontarget trials, as well as increased P3b amplitude and shortened P3b latency in target trials during the spatial 2‐back task.

**Conclusions:**

These results suggested that WM training might enhance children's behavioral performance on WM tasks and brought about neurophysiological changes. This study gives insights into the potential of WM training effects on children's behavioral performance and neurophysiological outcomes.

## Abstract 2

This work found that working memory training enhanced children's working memory performance and related ERP measures, providing evidence for the efficacy of working memory training on children.

## 1 INTRODUCTION

Working memory (WM) is defined as a cognitive system for the temporary maintenance and manipulation of information (Baddeley, 2012; Pergher et al., 2018), which has always been considered to play a significant role in many other cognitive operations, such as learning, reasoning, intelligence, and problem‐solving (Alloway, 2009; Baddeley, 2003; Conway et al., 2003).

Several recent studies and reviews have examined the effect of computerized cognitive training on WM but fail to reach any agreement (Backman et al., 2017; Pappa et al., 2020; Sala & Gobet, 2017; Sala et al., 2019). In these training studies, simple digit span tasks, complex span tasks, and N‐back tasks have generally been taken as training tasks (Hayashi, 2019; Pergher et al., 2018; Spencer‐Smith & Klingberg, 2015; Zhao et al., 2013). These tasks were considered to involve both short‐term memory storage component and the central executive component (Scharinger et al., 2017; Unsworth et al., 2009). But both the multi‐component model of WM and the attention control model suggested that the central executive component occupied a central position in WM (Baddeley, 2003, 2010, 2012; Unsworth et al., 2009), and shared the brain mechanism with other complex cognitive operations (Jung & Haier, 2007; Owen et al., 2005). It was the central executive component rather than storage component closely related to advanced cognitive functions (Conway et al., 2003). Thus, training tasks requiring the participation of central executive component, in our opinions, have the particularly important practical application value for the development of individuals. The training effects of N‐back tasks on central executive component have been confirmed by many studies (Ang et al., 2015; Jaeggi et al., 2011; Pergher et al., 2018). Also, as N‐back tasks could effectively induce electrical components and the activation of the cerebral cortex (Scharinger et al., 2017), these tasks have been the most commonly used experimental paradigms to investigate the neuronal connections between WM function and WM training effects (Chen et al., 2019; Owen et al., 2005; Schneiders et al., 2011). Although complex span tasks have long been generally regarded as containing both two WM components, recent research found that complex span tasks could induce WM manipulation‐load as effectively as N‐back tasks and show similar electroencephalogram (EEG) patterns. Thus, their important role in the training of central executive component should be given attention (Unsworth & Engle, 2005; Unsworth & Engle, 2008; Unsworth et al., 2009). To the best of our knowledge, there have been few studies that used both complex span task (i.e., computation‐span task) and N‐back task (i.e., spatial N‐back task) as central executive training tasks to examine the training effects on WM.

In terms of the effects of WM training, the results tended to be inconsistent (Pappa et al., 2020; Redick et al., 2015; Sala & Gobet, 2020). Some studies found that WM training could lead to performance improvement on untrained WM tasks (Henry et al., 2014; Redick et al., 2015; Sala & Gobet, 2020; Soveri et al., 2017). They suggested that personal WM was plastic and WM training might lead to improved WM function and better task performance in WM tasks, though the types of training tasks, modalities of stimulation, and the tasks used to evaluate training effects were different (Adam & Vogel, 2018; Gathercole et al., 2019; Hayashi, 2019; Sala & Gobet, 2020; Spencer‐Smith & Klingberg, 2015). On the contrary, other studies all found evidence of no performance improvement on untrained WM tasks after WM training (Backman et al., 2017; Linares et al., 2019). Thus, one of the objectives in the current study was to explore behavioral effects on untrained WM tasks following WM training.

In recent years, the development and application of cognitive neuroscience technology has provided new ideas for evaluating the effects of WM training (Buschkuehl et al., 2012). Neuroimaging technology has been used to explore neuronal effects of WM training, providing convincing evidence for the plasticity of WM (Constantinidis & Klingberg, 2016; Schneiders et al., 2011). The event‐related potentials (ERPs) technique has a high time resolution of microseconds and could accurately record ERPs on the time series of EEG signals. Thus, ERP has been considered as an important tool for examining the neuronal effects of WM (Scharinger et al., 2017; Zhao et al., 2013).

Among many components of ERP, P3 has long been considered to be closely related to WM (Dong et al., 2015; Lubitz et al., 2017). In accordance with the context updating model and resource allocation theory (Gajewski & Falkenstein, 2018; Zhao et al., 2013), P3 reflects processes of detection of stimuli changes, and establishes representations updating and cognitive resources allocation in mind (Polich, 2007; Scharinger et al., 2017). P3 amplitude is thought to represent the recruitment of neural resources for WM cognitive processes, while P3 latency is proved to be related to stimulus evaluation time, which reflects the speed of information processing (Kutas et al., 1977; Thompson et al., 2016). P3 component includes the activity of two ERP subcomponents, P3a and P3b (Polich, 2007). Specifically, P3a originates from frontal activity, and it is considered to be closely related to stimulus‐driven attention mechanisms and the processing of novel stimuli (Friedman et al., 2001; Gajewski & Falkenstein, 2018). P3b originates from temporal–parietal activity, and it is considered to be closely related to subsequent memory processing and operation. It reflects attention and cognitive resources allocation in mind (Gajewski & Falkenstein, 2018; Polich, 2007). The amplitudes and latencies of P3a and P3b actually to some extent represent the performance of the WM function (Covey et al., 2018; Pergher et al., 2018; Zhao et al., 2013). Well‐performing individuals are allowed to invest less cognitive processing resources and complete faster than underperforming individuals faced with the same task, and they have higher amplitudes and shorter latencies of P3a and P3b correspondingly (Dong et al., 2015; Fjell et al., 2007; Lubitz et al., 2017). Therefore, from a theoretical point of view, the neural effects of WM training would be manifested as significantly increased amplitudes and shortened latencies of P3a and P3b.

This view has been supported by some empirical studies (Emch et al., 2019; Heinzel et al., 2016; Pergher et al., 2018; Zhao et al., 2013). From the previous WM training research, the brain regions where P3 amplitude enhanced or P3 latency reduced after training mainly involved frontal lobe as well as parietal lobe (Chen et al., 2019; Covey et al., 2018; Gajewski & Falkenstein, 2018), that is, WM training led to increased amplitudes and shortened latencies of P3a and P3b. However, there are a few studies which obtained inconsistent results (Liu et al., 2016; Woltering et al., 2021). Two studies found that there was no convincing evidence that the WM training per se changes neural activation patterns of behavioral tasks in Attention Deficit and Hyperactivity Disorder (ADHD) adults (Liu et al., 2016; Woltering et al., 2021). Thus, it remains an open question whether WM training could lead to increased amplitudes and shortened latencies of P3a and P3b.

At the same time, previous researchers usually recruited adults and the elderly as the research objects to explore the WM training effects on brain activity (Chen et al., 2019; Nussbaumer et al., 2015; Pergher et al., 2018; Zhao et al., 2013), whereas few studies have been conducted on children. However, children represent a particularly important population on which to perform WM training (Sala & Gobet, 2017). They are just at the beginning of cognitive ability and academic skills development in which WM, especially the central executive component of WM, plays a fundamental role. Moreover, compare with the speed and stability of brain development in adults, children are in the stage of rapid development of cognitive function (Johnson, 2001; Spitzer, 2017). Thus, WM training on children seems to be efficient and far‐reaching. As training studies in children using ERP were few, the second objective of the current study was to investigate whether WM training could have an impact on the amplitudes and latencies of P3a and P3b in children with the purpose of supplementing previous studies.

In summary, the purpose of our study was to investigate the effects of WM training based on both the computation‐span task and spatial N‐back task in children, including the effects on both behavioral performance and neurophysiological outcomes. To investigate this, taking second‐ and third‐grade children as the participants, we tended to examine whether WM training could lead to behavioral improvement and changes in the amplitudes and latencies of P3a and P3b. It was hypothesized that we would observe the increased amplitudes and shorter latencies of P3a and P3b during the training process as well as the behavioral improvements in post‐test of the training group, which would suggest the potential of WM training effects on children's behavioral performance and neurophysiological outcomes.

## 2 METHODS

### 2.1 Participants

The participants were recruited on a voluntary basis from four classes in a public primary school in Shanghai, China. The participants were right‐handed and native Mandarin speakers, with normal or corrected vision, no color blindness or weakness, no history of mental illness or neurological diseases, no medical treatment before the measurements, and never participated in similar cognitive training. Sample size was determined by a power analysis prior to data collection using G*Power. Based on a conservative effect size in the small‐medium range (*ŋ_p_^2^* = 0.05), estimated from previous research on behavioral and neural effects of WM training (Pergher et al., 2018), we determined a target sample size of 40 participants. We collected as much data as we could, a total of 44 second‐ and third‐grade healthy children constituted the final experimental sample. All participants were randomly divided into two groups, the training group (*n* = 21, mean age = 7.73 years, *SD* = 0.59 years, 9 females) and the control group (*n* = 23, mean age = 7.79 years, *SD* = 0.56 years, 9 females). For an overview of the sample characteristics, see Table 1. They all completed the pre‐ and post‐test behavioral evaluation during the training process. A sensitivity power analysis for behavioral results conducted in G*Power (Faul et al., 2007; alpha = 0.05, power = 0.80, groups = 2) indicated that, given this sample size, the study was powerful enough to detect a medium effect size, *ŋ_p_^2^* = 0.07, *f *= 0.26 (Cohen, 2013). Unfortunately, as using ERP in children was difficult and extremely time‐consuming, EEG data of only 31 children could be collected before school holidays. Thus, all 21 children in the training group (mean age = 7.73 years, *SD* = 0.59 years, 9 females) and only 10 children in the control group (*n* = 10, mean age = 7.65 years, *SD* = 0.62 years, 4 females) with available EEG data were included in EEG data analyses. A sensitivity power analysis for ERP results conducted in G*Power (Faul et al., 2007; alpha = 0.05, power = 0.80, groups = 2) indicated that, given this sample size, the study was powerful enough to detect a medium effect size, *ŋ_p_^2^* = 0.06, *f *= 0.25 (Cohen, 2013). The study was approved by the Academic Ethics Committee of Shanghai Normal University. The legal guardians of all participants gave informed written consent before testing began. All the participants were given a present (i.e., pencil, ruler, notebook, etc.) as a compensation for their time and participation after the experiment.

**TABLE 1:** Demographic characteristics of the samples

	Training group	Control group
Age	7.73 (0.59)	7.79 (0.56)
Gender		
Female	9 (42.85%)	9 (39.17%)
Male	12 (57.14%)	14 (60.83%)
Grade		
Grade 2	11 (52.38%)	10 (43.47%)
Grade 3	10 (47.62%)	13 (56.53%)
Father's education		
High school and below	4 (19.05%)	2 (8.70%)
Junior college or college degree	12 (57.14%)	15 (65.22%)
Master's degree and above	5 (23.81%)	6 (26.07%)
Mother's education		
High school and below	2 (9.52%)	2 (8.70%)
Junior college or college degree	14 (66.67%)	18 (78.26%)
Master's degree and above	5 (23.81%)	3 (13.04%)

### 2.2 Measures

#### 2.2.1 Tests and materials for pre‐ and post‐test evaluation

Digit span backwards task and N‐back task were used as the measurement materials for WM of children (Rosen et al., 2020; Vuontela et al., 2003).

***Digit span backwards task***. This task adopted from the Wechsler Intelligence Scale for Children (WISC‐IV; Wechsler, 2003). Children were presented with sequences of digits and required to repeat the sequences backward. The strings of numbers were read aloud by the experimenter at a rate of one digit per second. The task started with two digits. If the participants recalled correctly, the length of the sequence increased by one digit, the maximum length was nine digits. Each length consisted of four trials. The task ends when the participant fails in all four attempts of the given number length. The longest correct sequences of digits the participants achieved was used as the participant's score in this task.

***N‐back task***. In this task, E‐Prime 1.1 was used to present stimuli and collect data. This study used N‐back task (1‐back and 2‐back) with different memory load level, and asked the participants to match the current digit with the digit in front of the N positions, accurately and quickly judge whether they were the same. In the 1‐back task, participants were asked to determine whether the current displayed digit was the same as the digit in front of the 1 position; in the 2‐back task, participants were asked to compare whether the current digit was the same as the digit in front of the two positions. The 1‐back and 2‐back tasks each consisted of 24 trials, half of which were target trials and the other half were nontarget trials. A target appeared when the current digit was identical to the digit shown before. Similarly, a nontarget appeared when the current digit was different to the digit shown before. Participants were instructed to press the key “A” on the keyboard within a 3500 ms time limit for the targets, otherwise they needed to press the key “L.” The accuracy (i.e., correct responses as a percentage of the total trials) was used as the participant's score in the 1‐back and 2‐back tasks, respectively.

#### 2.2.2 Training tasks

Training tasks included computation‐span task and spatial N‐back task (Minear et al., 2016).

**FIGURE 1:**
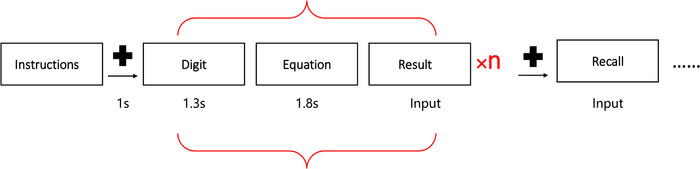
Graphical rendition of computation‐span task

***Computation‐span task***. The training program was compiled with E‐prime 1.1. The stimuli were presented in the center of the screen and the materials appeared in random order. The participants were asked to memorize the digits presented in order and perform mathematical equations between the digit presentations (**Figure** **1**). The participants saw a digit in the center of the screen at first, a mathematical equation would be presented when the digit disappeared. Then, the participants required to solve the mathematical equation and input the result using the keyboard in the following screen. After that, the participants would see another digit. After a certain number of combinations of digits and mathematical equations appearance, a screen will be presented to remind participants of recalling, and participants need to input digits in the order presented previously. The participants had to complete eight sets of computation span trials in every session associated with a different difficulty level. There were four levels of difficulty in the task which was determined by the number of digits requiring to be recalled in a trial. Training score was the percentage of correct recalled trials. The participants started training from a low span level (*n* = 3), and when their completion accuracy reached 85%, they would be allowed to enter the next difficulty level.

**FIGURE 2:**
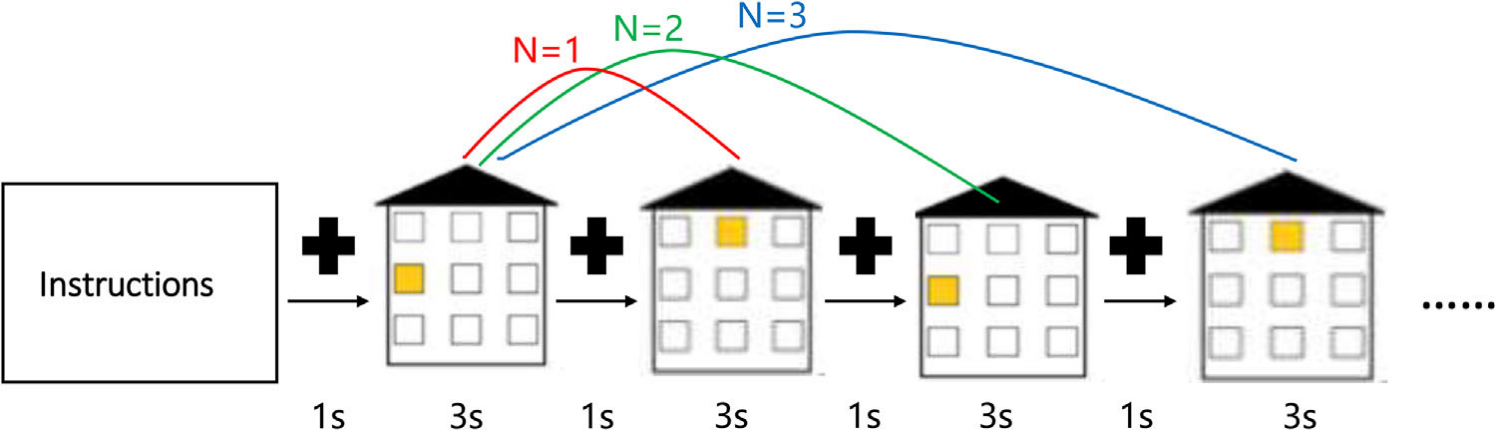
Graphical rendition of special N‐back task

***Spatial N‐back task***. The participants were asked to memorize and recognize the constantly refreshing visual spatial stimuli, comparing the stimuli information currently presented with the stimuli in front of the *N* positions and making responses with the keyboard (**Figure** **2**). The stimulation of the training task used randomly presented pictures with spatial information. In order to make it easier for elementary school students to understand, we chose pictures of houses and windows. A house with nine windows was presented on the screen. Each time the house appeared, one of the windows will be lit. The participants were asked to remember which window was lit, and compare whether the current displayed lit window on the screen is in the same position as the *N* lit windows before (*N* = 1 in 1‐back; *N* = 2 in 2‐back; and *N* = 3 in 3‐back). When spatial information currently presented was completely consistent with the information in the *N* positions ahead, the participants were asked to press the “A” on the keyboard (33% of the pictures are the same target), otherwise press the “L” on the keyboard. The participants had to complete 60 trials in each session which was associated with a level of difficulty. There were three levels of difficulty in the task, which is determined by the *N* position, they are 1‐back task, 2‐back task, and 3‐back task. The training task score was the percentage of the trials that participants answered correctly. Participants started training at a low difficulty level (1‐back), and when the accuracy rate reached 85%, they could enter the next difficulty level (2‐back and 3‐back).

### 2.3 Procedure

**FIGURE 3:**
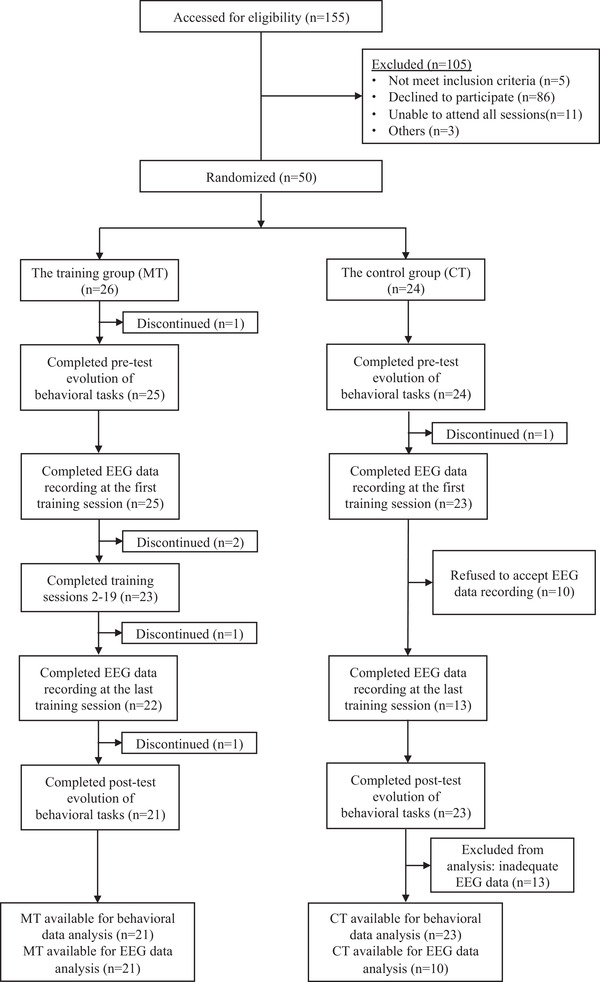
CONSORT flow chart of participants through the study

Subject flow is presented in **Figure** **3** based on the CONSORT reporting instructions (Schulz et al., 2010). Participants in the training group completed 20 training sessions in school that were preceded by a pre‐test evaluation and followed by a post‐test evaluation, while the control group was involved in normal class activities and conducted no training between the pre‐test and post‐test evaluation in order to examine the training effects on children's behavioral performance (pre‐ to post‐test days elapsed: *M* = 11.72 months, *SD* = 0.35 months).

Only if the accuracy rate reached 85%, participants could enter the next difficulty level (2‐back and 3‐back). Few participants could use the spatial 3‐back task for training at the beginning. As a result, EEG data were collected twice to analyze the training effects during WM training process, respectively, at the first and last training session, using the spatial 1‐back and 2‐back tasks. For the control group, EEG data collection was conducted with the same training task and at the same time point as the training group. Forty‐four children all completed the pre‐ and post‐test behavioral evaluation as well as EEG data collection during the training process. Unfortunately, all 21 children in the training group and only 10 children in the control group with available EEG data were included in EEG data analyses as using ERP in children was difficult and extremely time‐consuming.

**FIGURE 4:**
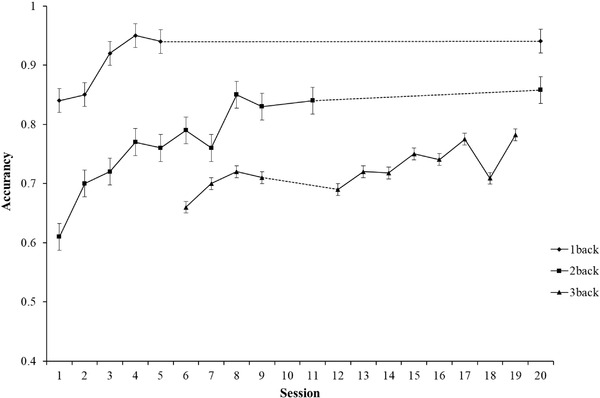
Spatial N‐back performance across every session

**FIGURE 5:**
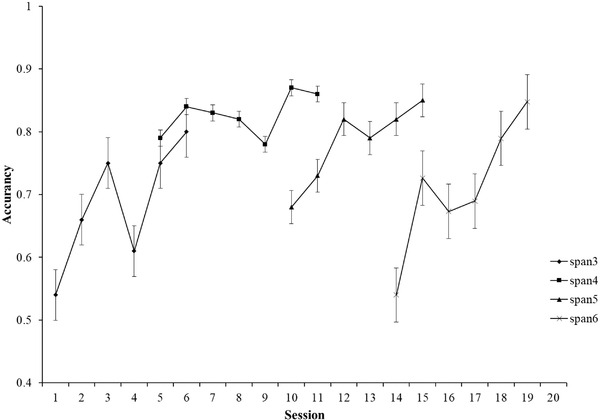
Computation‐span task performance across every session

Within the training group, participants were instructed to perform the spatial N‐back task and the computation‐span task in order in each training session. Participants completed 20 sessions of training, for 2–3 times a week (about 30 min each). The interval between each training session was 2–3 days, which was determined based on the participants’ daily class schedule. There were three difficulty levels in the spatial N‐back task (i.e., 1‐, 2‐, and 3‐back) and four difficulty levels in the computation‐span task (i.e., span = 3, 4, 5, and 6). The training process started with tasks with low cognitive load or small span. When the correct rate of student task completion reached more than 85%, they entered a higher difficulty level of training tasks. Twenty sessions of training for 21 children in the training group were completed within 5.5 months. All the evaluation and training tasks were conducted by professionally trained graduate students. For the specific situation of 20 training sessions, see Figures 4 and 5.

### 2.4 EEG data recording and offline processing

EEG data collection was conducted in a closed room with soft light and quietness. After the participants were seated in comfortable chairs, the electrode caps were tightly and properly worn on the participants' heads. The participants were guided to adjust their postures, with their eyes about 1 m away from the computer screen. In order to minimize the interference of artifacts, the participants were required to keep their bodies immobile and reduce the number of blinks throughout the experiment.

EEG activity was recorded continuously with SynAmps amplifiers from 32 Ag/AgCl electrodes using a 32‐channel cap (10−20 system) of a NeuroScan system (Neuro Scan Inc. El Paso, TX, USA). The reference electrodes were positioned at the left and right mastoids and the ground electrode was located at FPz. The horizontal electrooculogram (EOG) was recorded throughout two electrodes positioned at the external canthi of both eyes, and the vertical EOG was recorded throughout two electrodes positioned above and below the left eye. All signals were digitized at a sampling rate of 500 Hz. During the EEG recording, electrode impedances were maintained below 5 kΩ.

EEG data offline analysis was conducted by custom scripts combined with functions in EEGLAB (Delorme & Makeig, 2004) and ERPLAB (Lopez‐Calderon & Luck, 2014) under MATLAB (R2017b) software. To remove high‐ and low‐frequency noise, the EEG data were cleaned using a bandpass filter (0.1‐40 Hz, IIR Butterworth, second order) by *pop_basicfilter* function in ERPLAB. Next, in order to identify and remove flatline channels, low‐frequency drifts, and noisy channels, *clean_artifacts* function in EEGLAB was employed; meanwhile, transient or large‐amplitude artifacts were corrected using artifact subspace reconstruction method implemented in *clean_artifacts* function (Chang et al., 2020). Then, independent component analysis was performed, and the artifact components, including eye, muscle, heart, line noise, and channel noise, were identified by ICLabel in EEGLAB and rejected from the EEG data. After that, the rejected bad channels were interpolated by spherical method. EEG data were then segmented into epochs of 1000 ms, which included the 200‐ms prestimulus baseline period. The epochs with the maximum peak‐to‐peak voltage above 100 μV at channels Fz and Pz were rejected using moving window peak‐to‐peak threshold method in ERPLAB. The two groups did not have significant difference in artifacts‐free trial counts in 1‐back (the control group: *M *= 25.63, *SE *= 0.98; the training group: *M *= 25.22, *SE *= 0.68, *p *= .741) and 2‐back (the control group: *M *= 24, *SE *= 1.26; the training group: *M *= 23.93, *SE *= 0.87, *p* = .963).

ERP measurement function *pop_geterpvalues* in ERPLAB was utilized to extract ERP features, where local peak option was utilized as this method can prevent the rising edge of an adjacent component at the edge of the measure window from being chosen as the peak (Lopez‐Calderon & Luck, 2014). Based on the previous research (Covey et al., 2018; Gajewski & Falkenstein, 2018), P3a amplitude was quantified as local peak amplitude and its latency in the 300–600 ms time window at Fz. Similarly, the amplitude and latency of P3b were quantified as local peak amplitude and its latency in the 300–600 ms time window at Pz. Therefore, we mainly analyzed the peak amplitudes and latencies of P3a and P3b in the 300‐600 ms time window in the spatial 1‐back and 2‐back tasks, focusing on the midline electrodes in the frontal and parietal regions (i.e., Fz and Pz).

### 2.5 Statistical analyses

For the analysis of behavioral data, we conducted several repeated‐measures ANOVAs with session (pre‐test and post‐test) as the within‐subjects factor, group (the training group and the control group) as the between‐subjects factor, and the scores on WM tasks as the dependent variables.

For the analyses of ERP data, based on the previous research (Covey et al., 2018; Gajewski & Falkenstein, 2018), in which they conducted group × session × trial type repeated measures ANOVAs for the computerized tests that had multiple trial types (i.e., target and nontarget), we analyzed target‐locked and nontarget‐locked ERPs separately to reduce complexity of the data. The amplitudes and latencies of P3a and P3b were analyzed using a three‐way ANOVA (group × session × trial type), with session (the first training session and the last training session) and trial type (target trials and nontarget trials) as the within‐subjects factors, group (the training group and the control group) as the between‐subjects factor. Following the previous research (Zhang et al., 2019), we calculated effect sizes in all ANOVAs to minimize the effect of unbalanced sample size.

The statistical analysis was conducted by IBM SPSS 25.0.

## 3 RESULTS

### 3.1 Effects of WM training: Behavioral results

Descriptive statistics (means and standard deviation) of each WM task performance are presented in **Table** **2** for the training group and control group, for pre‐and post‐tests. We found no significant differences between the training group and control group in pre‐test performance (*t *= 0.24−1.32, *ps* > .05).

**TABLE 2:** Descriptive statistics for each WM task performance *M* (*SD*)

	Training group (*n* = 21)		Control group (*n* = 23)	
	Pre‐test	Post‐test	Pre‐test	Post‐test
Digit span backwards	4.76 (1.61)	6.19 (1.08)	4.65 (1.43)	5.13 (1.46)
1‐back	0.82 (0.16)	0.96 (0.04)	0.75 (0.18)	0.85 (0.15)
2‐back	0.55 (0.16)	0.83 (0.14)	0.52 (0.19)	0.58 (0.17)

Results of repeated‐measures ANOVAs between group (the training group and the control group) and session (pre‐test and post‐test) are presented in Table 3.

**TABLE 3:** Results of repeated‐measures ANOVAs between group (the training group and the control group) and session (pre‐test and post‐test)

	Session (S)			Group (G)			G × S		
	*F*	*p*	*η_p_^2^*	*F*	*p*	*η_p_^2^*	*F*	*p*	*η_p_^2^*
Digit span backwards	13.42	.00	0.24	3.02	.09	0.07	3.33	.08	0.07
1‐back	20.73	.00	0.33	6.30	.02	0.13	0.61	.44	0.01
2‐back	27.96	.00	0.40	12.66	.00	0.23	12.24	.00	0.23

For the digit span backwards task, results revealed a main effect of session, *F*(1, 42) = 13.42, *p *< .001, *ŋ_p_^2 ^* = 0.24. The effect of group was marginally significant, *F*(1, 42) = 3.02, *p *< .10, *ŋ_p_^2 ^* = 0.07. The interaction between group and session was also marginally significant, *F*(1, 42) = 3.33, *p *< .10, *ŋ_p_^2 ^* = 0.07. Further simple‐effects analysis showed that the score in the task significantly improved in the training group after training, *F*(1, 42) = 14.41, *p *< .001, *ŋ_p_^2 ^* = 0.26, but there was no significant change in the control group, *F*(1, 42) = 1.77, *p* = .19.

For the 1‐back task, results revealed a main effect of session, *F*(1, 42) = 20.73, *p *< .001, *ŋ_p_^2 ^* = 0.33. The effect of group was significant, *F*(1, 42) = 6.30, *p *< .05, *ŋ_p_^2 ^* = 0.13. The interaction between group and session was not significant, *F*(1, 42) = 0.61, *p *= .44.

For the 2‐back task, both the effect of session, *F*(1, 42) = 27.96, *p *< .001, *ŋ_p_^2 ^* = 0.40, and group, *F*(1, 42) = 12.66, *p *< .001, *ŋ_p_^2 ^* = 0.23, reached significance. Moreover, an interaction between group and session was found, *F*(1, 42) = 12.24, *p* < .01, *ŋ_p_^2 ^* = 0.23. Further simple‐effects analysis showed that the accuracy in the 2‐back task significantly improved in the training group after training, *F*(1, 42) = 36.92, *p *< .001, *ŋ_p_^2 ^* = 0.47, but there was no significant change in the control group, *F*(1, 42) = 1.68, *p* = .20.

### 3.2 Effects of WM training: ERP results

**FIGURE 6:**
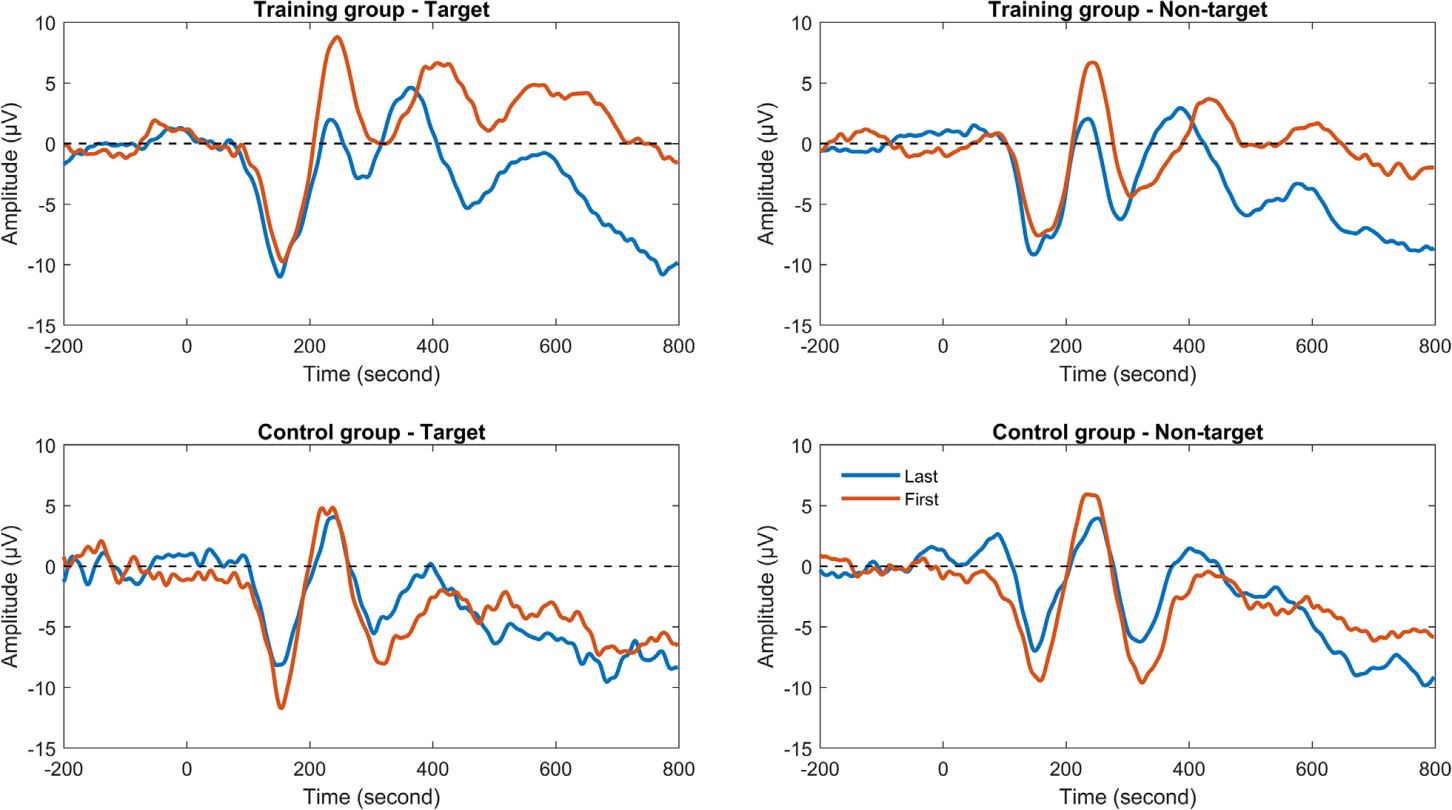
P3a in target and nontarget trials of the spatial 1‐back task for the training and control groups

Figure 6 shows grand average of P3a in target and nontarget trials of the spatial 1‐back task for the training and control groups. For P3a amplitude, results from ANOVA showed that there were no significant main effects and interactions. For P3a latency, there was a significant group × session × trial type interaction, which meant significant differences between target and nontarget trials for the interaction between group × session, *F*(1, 29) = 8.83, *p *< .01, *ŋ_p_^2 ^* = 0.23. Further analysis showed that the group × session interaction in target trials was nonsignificant, *F*(1, 29) = 0.19, *p* = .66, while the interaction in nontarget trials was significant, *F*(1, 29) = 12.73, *p *< .01, *ŋ_p_^2 ^* = 0.31. Simple‐effects analysis showed that P3a latency in nontarget trials was shortened in the training group after training, *F*(1, 29) = 24.24, *p *< .001, *ŋ_p_^2 ^* = 0.46, but there was no change in the control group, *F*(1, 29) = 0.88, *p* = .36.

**FIGURE 7:**
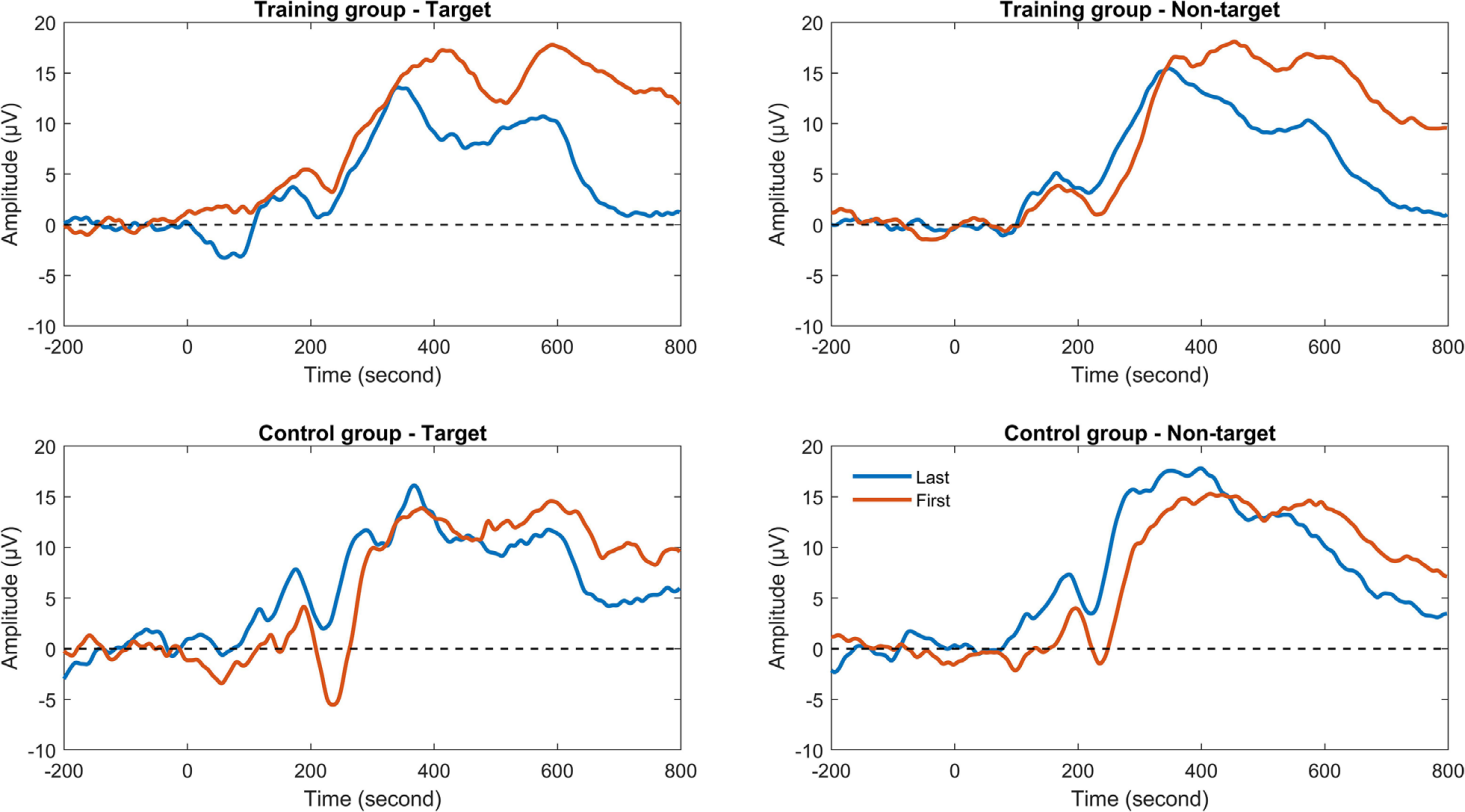
P3b in target and nontarget trials of the spatial 1‐back task for the training and control groups

Figure 7 presents grand average of P3b in target and nontarget trials of the spatial 1‐back task for the training and control groups. For P3b amplitude, results from ANOVA showed that there were no significant main effects and interactions. For P3b latency, there was a significant group × session × trial type interaction, which meant significant differences between target and nontarget trials for the interaction between group × session, *F*(1, 29) = 5.19, *p *< .05, *ŋ_p_^2 ^* = 0.15. Further analysis showed that the group × session interaction in target trials was nonsignificant, *F*(1, 29) = 0.17, *p* = .69, while the interaction in nontarget trials was significant, *F*(1, 29) = 5.97, *p *< .05, *ŋ_p_^2 ^* = 0.17. Simple‐effects analysis showed that P3b latency in nontarget trials was shortened in the training group after training, *F*(1, 29) = 6.15, *p *<.05, *ŋ_p_^2 ^* = 0.18, but there was no change in the control group, *F*(1, 29) = 1.58, *p* = .22.

**FIGURE 8:**
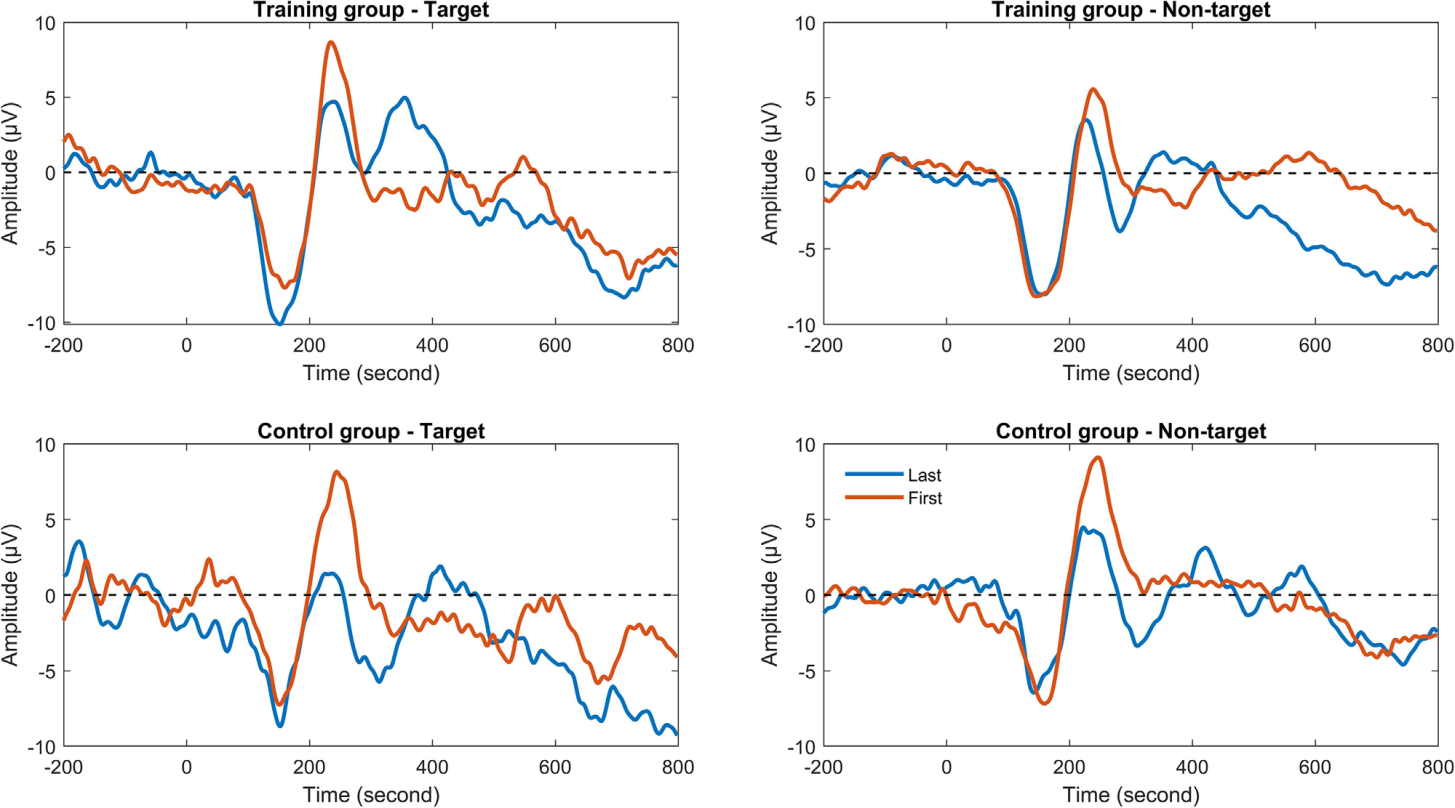
P3a in target and nontarget trials of the spatial 2‐back task for the training and control groups

Figure 8 depicts grand average of P3a in target and nontarget trials of the spatial 2‐back task for the training and control groups. For P3a amplitude, results from ANOVA showed that there were no significant main effects and interactions. For P3a latency, an interaction between group and session was found, *F*(1, 29) = 8.84, *p* < .01, *ŋ_p_^2 ^* = 0.23. Further simple‐effects analysis showed that P3a latency was shortened in the training group after training, *F*(1, 29) = 18.00, *p *< .001, *ŋ_p_^2 ^* = 0.38, but there was no change in the control group, *F*(1, 29) = 0.47, *p* = .50.

**FIGURE 9:**
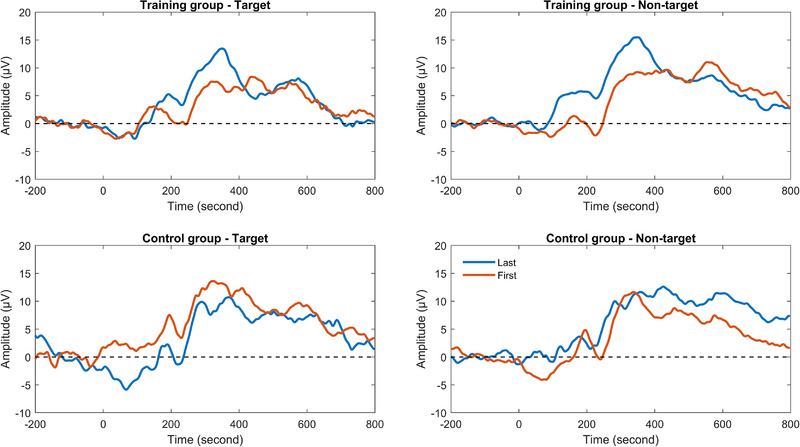
P3b in target and nontarget trials of the spatial 2‐back task for the training and control groups

Figure 9 shows grand average of P3b in target and nontarget trials of the spatial 2‐back task for the training and control groups. For P3b amplitude, there was a trend toward group × session × trial type interaction, which meant marginally significant differences between target and nontarget trials for the interaction between group × session, *F*(1, 29) = 3.79, *p *< .10, *ŋ_p_^2 ^* = 0.12. Further analysis showed that the group × session interaction in target trials was significant, *F*(1, 29) = 5.53, *p *< .05, *ŋ_p_^2 ^* = 0.16, while the interaction in nontarget trials was nonsignificant, *F*(1, 29) = 0.01, *p* = .93. Simple‐effects analysis showed that P3b amplitude in target trials was marginally improved in the training group after training, *F*(1, 29) = 3.15, *p *< .10, *ŋ_p_^2 ^* = 0.10, but there was no change in the control group, *F*(1, 29) = 2.67, *p* = .11. For P3b latency, an interaction between group and session was found, *F*(1, 29) = 6.70, *p* < .05, *ŋ_p_^2 ^* = 0.19. Further simple‐effects analysis showed that P3b latency was marginally shortened in the training group after training, *F*(1, 29) = 3.95, *p *< .10, *ŋ_p_^2 ^* = 0.12, while P3b latency of the control group was marginally lengthened, *F*(1, 29) = 3.14, *p *< .10. *ŋ_p_^2 ^* = 0.10.

## 4 DISCUSSION

As behavioral and neural effects of WM training studies were controversial, we aimed to examine whether WM training could lead to the increased amplitudes and shortened latencies of P3a and P3b during the training process and a significant behavioral improvement in WM tasks of post‐test. To investigate this, we conducted a study where 21 children in the training group and 23 children in the control group were assessed their behavioral performance improvement on a battery of WM tasks (digit span backwards and N‐back tasks) before and after 20 training sessions, and they completed their EEG recordings, respectively, at the first and last spatial N‐back task (1‐ and 2‐back) training sessions to analyze effects on ERP components (31 children with available EEG data were included in EEG data analyses).

In terms of behavioral performance, we found that WM training based on both the complex span tasks and spatial N‐back tasks improved some post‐test WM tasks performance of participants in the training group compared to the control group. More specifically, the training effects appeared as the improvements of the digit span backwards task and 2‐back task in the training group, which was consistent with previous research results (Banales et al., 2015; Etherton et al., 2019; Peng et al., 2017). There was no significant difference, however, in the 1‐back task between pre‐test and post‐test of the training and control groups. One possible reason might be that 1‐back task had a ceiling effect. It has been confirmed by many studies that 2‐back task was effective to induce a greater WM load for central executive component of WM to explain the WM training effects more intuitively (Davidson et al., 2018; Peng et al., 2013). Thus, it was naturally not surprising that WM training brought a significant performance improvement in the digit span backwards task and 2‐back task.

As for ERP, P3a and P3b components of the training group showed some changes from the first to the last training sessions: shortened P3a and P3b latencies in nontarget trials during the spatial 1‐back task; shortened P3a latency in target and nontarget trials; and increased P3b amplitude and shortened P3b latency in target trials during the spatial 2‐back task. This result supported the behavioral improvement of WM tasks to a certain extent, suggesting WM training effects on children's neurophysiological outcomes.

In the spatial 1‐back task, the training effects were only reflected in shortened P3a and P3b latencies in nontarget trials, which meant that training to a certain extent speed up the information processing speed for stored items. However, we did not find the change of P3a and P3b latencies in target trials as well as P3a and P3b amplitudes, which implied that the neural effects of WM training on the spatial 1‐back task were relatively limited. This result supported the finding of the 1‐back task behavioral performance to a certain extent. Also, this result was consistent with some previous studies (Pergher et al., 2018; Tusch et al., 2016).

Compared with the spatial 1‐back task, the neural training effects on the spatial 2‐back were more obvious. Shortened P3a in target and nontarget trials, increased P3b amplitude, and shortened P3b latency in target trials during the spatial 2‐back task supported the finding of previous studies that P3 components seemed to play a more important role in 2‐back paradigm (Pergher et al., 2018; Tusch et al., 2016). The decreasing trend of P3a latency not only appeared in target trials, but also in nontarget trials, suggesting increase in the attention maintenance and processing speed of novel stimuli. In contrast to the control group, increased P3b amplitude and shortened P3b latency in target trials suggested reduced occupation and efficient distribution of cognitive resources as well as faster cognitive processing speed when faced with the same cognitive load task. These results supported the finding of behavioral performance in the 2‐back task to a certain extent.

Several limitations of the current study should be considered. First, the sample size of the training group (*n* = 21) and the control group (*n* = 10) of EEG data was unbalanced as using ERP in children was difficult and extremely time‐consuming. The consequence of imbalance was the reduced design power, therefore, it was particularly possible to overlook an effect (i.e., judge it as not significant) when the effects truly existed (Hector et al., 2010; Shaw & Mitchell‐Olds, 1993). However, the observed training effects in EEG were significant in this study. Considering the reduced design power caused by unbalanced sample size, EEG results which showed significant changes with 20 training sessions were convincing to a certain extent, providing potential neurophysiological evidence for behavioral performance improvement. Nevertheless, the results should be interpreted with caution, and the findings should be replicated in a larger and balanced sample in future research. In addition, no placebo group was used in this study, thus, positive experimental results generated by the psychological effect of self‐suggestion of the participants could not be avoided. Moreover, another important indicator of the effectiveness of WM training is the transfer effects, which meant that training‐related benefits in different cognitive functions will be obtained in addition to WM, and the future research should focus on the transfer effects on other cognitive operations of WM training and attempt to explain it in terms of brain mechanism. Last but not least, although the ERP technique has high time resolution, its spatial resolution is relatively low. It is an irreversible trend to combine ERP and other neuroimaging techniques, such as fMRI or fNIRS, to accurately record the time course and precisely locate the brain activity in certain areas in future studies.

In general, the research results are basically consistent with our assumptions, the better behavioral performance of children in the digit span backwards task and 2‐back task of post‐test evaluation, higher P3b amplitude, and shorter P3a and P3b latencies prove the behavioral and neural effects of WM training, suggesting that WM training might not only improve children's behavioral performance on WM tasks, but also brought about neurophysiological changes.

## 5 CONCLUSIONS

This study aimed to investigate the effects of WM training in children, including the effects on both behavior performance and neurophysiological outcomes. The results showed that WM training led to improved performance in the digit span backwards task and 2‐back task of post‐test evaluation, as well as change of ERP components in the spatial N‐back task. These results give insights into both the potential of WM training effects on children's behavioral performance and neurophysiological outcomes.

## Data Availability

The data that support the findings of this study are available on reasonable request from the corresponding author. The data are not publicly available due to privacy or ethical restrictions.
